# Experimental and Computer Simulation Studies on Badminton Racquet Strings

**DOI:** 10.3390/s23135957

**Published:** 2023-06-27

**Authors:** Narakorn Suwannachote, Thanongsak Imjai, Chirawat Wattanapanich, Fetih Kefyalew, Reyes Garcia, Pakjira Aosai

**Affiliations:** 1 School of Languages and General Education, Walailak University, Nakhon Si Thammarat 80160, Thailand; snopphor@wu.ac.th; 2 School of Engineering and Technology, Walailak University, Nakhon Si Thammarat 80160, Thailand; wchirawa@wu.ac.th (C.W.); fetihkefyalew.te@mail.wu.ac.th (F.K.); pakjira.ao@mail.wu.ac.th (P.A.); 3 Civil Engineering Stream, School of Engineering, University of Warwick, Coventry CV4 7AL, UK; reyes.garcia@warwick.ac.uk

**Keywords:** badminton racquet, shuttle cock, natural frequency, finite element analysis, impact

## Abstract

This study investigates experimentally, numerically, and analytically the performance of different string materials (Kevlar, synthetic gut, natural gut, and polyester) on badminton racquets. Vibration and impact tests with a shuttlecock were performed using a racquet frame made of carbon graphite mixed with epoxy resin. Different string tensions were considered in the tests (20, 22, 24, 28, 30, and 34 lb), as well as different hitting locations on the racquet frame. The results show that, as the diameter of the strings increased, the elasticity of the string decreased from 0.529 to 0.447 for diameters ranging from 0.62 to 0.70 mm. Subsequently, a badminton racquet and shuttlecock were modeled using SolidWorks2018^®^ software (version 26), and a maximum displacement was applied to the ball to simulate an impact on the string bed. The natural frequency, maximum deformation and maximum stress were calculated analytically, and a finite element analysis was also performed using ANSYS2022 R2^®^ software (version 22.2). The analytical and numerical results from ANSYS^®^ showed good agreement (within 5% accuracy). The results of the study show that the natural frequency of a racquet with Kevlar strings was significantly higher than that of racquets with synthetic gut, natural gut, or polyester string materials. Specifically, the natural frequency of a racquet made of carbon graphite and epoxy resin was 23.0%, 30.7%, and 36.2% higher than that of racquets with synthetic gut, natural gut, and polyester string material, respectively. On the basis of this finding, Kevlar was chosen as the preferred material for badminton racquets strings, and a parametric analysis was then conducted. The study showed that slightly lowering the tension of the off-centered strings had a minimal effect on the von Mises stress distribution of the ball and string bed. In addition to investigating string materials, this study also examined the effects of pull and diameter variations of racquet strings on vibrations during impact. This study contributes to the understanding of the role of racquet and strings in badminton, and it also provides new insights into the factors that can affect performance in the sport. By analyzing the performance of different string materials and examining the effects of pull and diameter variations of racquet strings, this study provides valuable information for players and manufacturers looking to optimize their equipment for maximum performance.

## 1. Introduction

Badminton is widely regarded as the fastest ball game in the world [1], with its pace primarily influenced by the introduction of new materials [2]. However, there is still a lack of understanding regarding the impact of various parameters on racquet strength, such as weight distribution, type of badminton racquet, and string tension [3]. Manufacturers rely on player feedback and experience rather than scientific methods to improve their racquet designs [4]. This trial-and-error approach does not guarantee optimal results and may occasionally lead to failures or racquets during matches. A more accurate development process could be achieved by gaining a deeper scientific understanding of the parameters that affect racquet performance and personalizing racquets accordingly. This is particularly valuable for manufacturers of high-performance racquets seeking to provide players with optimized equipment while enhancing performance and reducing injuries, which are the primary goals of athletes and coaches. Sports biomechanics research plays a crucial role in achieving these objectives [5].

Badminton racquets with greater weight generate higher ball speed in the smash than light racquets. This is due to the acceleration of angular and torque forces being higher in heavy racquets [6,7]. Research has shown that the speed of the shuttlecock is influenced by the tension of the strings. Higher string tension results in lower shuttlecock speed, as demonstrated by tests with variations in string pull of 22, 24, 26, 28, and 30 lb/ft^2^ [7,8]. These findings confirm that higher string tension leads to a lower rebound speed compared to lower string tension [8,9]. The diameter and tension of racquet strings vary on the basis of players’ preferences and needs. Previous research indicates that higher string tension results in a lower shuttlecock reflection [8]. The optimal string tension depends on the strength of the racquet frame, as excessively high tension can cause string breakage or warping, leading to breakage of the racquet frame. The strength and ball control achieved through string tension depend on the coefficient of friction on the string. String tension and friction coefficient are influenced by the quantity and frequency of stringing [10]. In the past, badminton racquets were constructed by attaching a net made of parchment or catgut to a wooden frame [11]. Over time, the sport has evolved into a fast-paced game, primarily due to advancements in racquet design. Modern racquets are primarily made of composite materials [2], enabling a stiff and lightweight construction. The strings are usually composed of a polymer that can withstand high tension, typically ranging from 90 to 140 N [12].

Modal response analysis, which involves evaluating the eigen-frequencies, can provide insights into racquet behavior. Each mode oscillates at a different frequency, and their combination influences the response during a hit. Furthermore, each mode has a unique shape that affects the subjective feeling when using the racquet. Li et al. [13] suggested that it is preferable to design tennis racquets to achieve the lowest bending mode, which includes the highest amplitude and energy. They also found that hitting the “dead spot” where the rebound effects are the lowest provides peak feedback for players. Hence, the vibrations of racquets play a significant role in a player’s judgment of a correctly performed stroke. Understanding the characteristics of a racquet, including its vibration, requires evaluating the influence of different parameters using eigen modes. The deflection behavior of the string is also considered one of the factors contributing to badminton’s reputation as the fastest ball sport [14].

The projectile used in badminton is referred to as a “shuttlecock”, and it consists of two important components: the feather cone and the sturdy cork base. The feather cone structure can be crafted from real feathers or from more resilient plastic materials. Although real feathers are costlier compared to synthetic alternatives, they offer greater consistency in flying characteristics [15], making them the preferred choice in competitive play. The shuttlecock’s design ensures aerodynamic stability with high drag properties, which is crucial for players to effectively strike the moving projectile. It has been found that shuttlecocks can reach a maximum speed of 113 m/s [16]. However, it has been observed that the racquet string tension also influences shuttlecock’s speeds. Vanasant et al. [7] reported that a high string tension (280 N) results in a lower rebound speed compared to a low string tension (180 N) in tennis balls. Moreover, it is generally accepted that, within the commonly used range of string tensions, a lower tension leads to higher rebound speed [9], whereas higher tensions aid in control. String tension does not only change the speed. Indeed, different string tensions can also influence a racquet’s flexibility, thus affecting speed and other impact-related factors [17]. When selecting a string tension, most players rely on recommendations from fellow players or on their personal preferences. According to a previous experimental study [9], only 27% of athletes were able to distinguish differences in string tension at 11 lb/ft^2^ (approximately 5 kg) while wearing earmuffs, and only 37% could differentiate between string tensions at 22 lb/ft^2^ (about 10 kg). If the string tension is too low, a significant amount of energy is absorbed, resulting in a lower rebound height [18].

Despite the popularity of badminton, there is still a research gap when it comes to understanding the effect of nonuniform string materials on racquet performance. Previous studies have focused on the effect of string tension and the type of string material on racquet performance, but the impact of variable diameter pull and nonuniform string materials remains unclear. To address this research gap, this study aims to analyze the pull and diameter of badminton racquet strings and their impact on vibration when in contact with the shuttlecock. By examining these variables, the study aims to help players select badminton racquet strings and pull that correspond to their needs in the game of badminton. The experimental and numerical simulation results from this study provide valuable data for performance enhancement by enabling athletes to select string tensions that match their playing style and needs. The ultimate objective of this research is to add a scientific perspective to the design of badminton racquets by studying the behavior during and after the collision between the badminton racquet string and the shuttlecock. This can provide designers and manufacturers with a better understanding of how nonuniform string materials affect racquet performance and how they can optimize the design of badminton racquets to enhance a player’s performance. The findings from this study can also contribute to the development of new technologies and materials for badminton racquets, improving their overall performance and making the sport more exciting and competitive for players and fans alike.

## 2. Experimental Program

### 2.1. Badminton Racquets and Shuttlecocks

**Figure 1:**
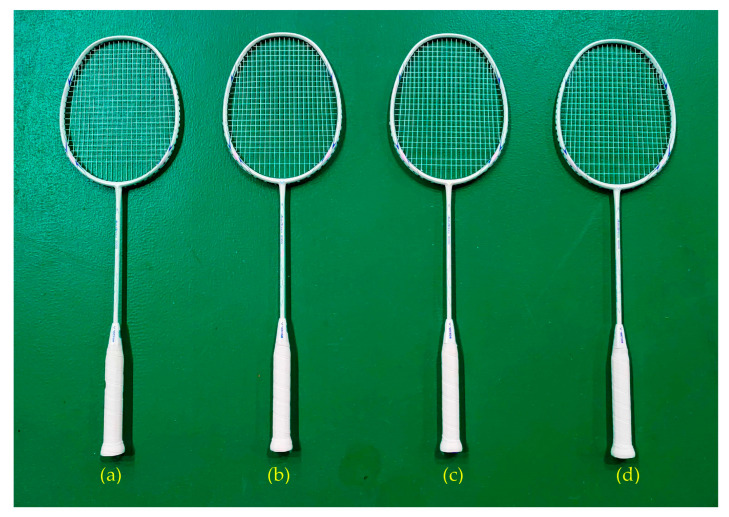
Carbon graphite mixed epoxy resin badminton frame (Victor model ARS-6000A) with different string patterns: (**a**) type A; (**b**) type B; (**c**) type C; (**d**) type D.

In this study, Victor racquets model AS 6000A are used in the tests, as shown in Figure 1. The racquet’s frame material is carbon graphite combined with resin, while the shaft material is made of carbon graphite + resin + 7.0 shaft. The string tension is ≤30 lb (13.5 kg per string), and the weight/grip size is 4U/G5. The graphite + resin frame material is a popular choice among badminton players because it offers a good balance of stiffness and flexibility. This also allows for better control and power during play. The addition of the 7.0 shaft enhances the racquet’s stability and reduces the chance of twisting during shots. The recommended string tension of ≤30 lb is suitable for players who want a balance of power and control. A higher string tension can increase power but may reduce control, while a lower string tension can increase control but may reduce power. The 4U weight and G5 grip size are also important factors to consider when selecting a badminton racquet. The 4U weight is a popular choice for players who want a lightweight racquet that is easy to maneuver, while the G5 grip size is suitable for players who prefer a thinner grip. Overall, the Victor AS 6000A is a high-quality badminton racquet that offers a balance of power, control, and maneuverability. Its graphite + resin frame material, 7.0 shaft, recommended string tension, 4U weight, and G5 grip size make it a great choice for intermediate to advanced players who want to improve their game.

This study investigated the effects of two variables on the vibration of badminton racquets. The independent variables in this study were the diameter and tension of the pull strings, while the dependent variable was the vibration of the shuttle racquet. The preparation phase of this study involved the use of six Victor racquets (Model ARS-6000A), Yonex shuttlecock (AEROSENSA 50) and Yonex strings (BG series). For the experimental tests, badminton racquets with Kevlar strings were utilized, following the results obtained from the numerical study outlined in Section 5.2. The racquets were strung with different tensions using an automatic machine to achieve 20, 22, 24, 28, 30, and 34 lb/ft^2^ in the pull strings, while the diameter of the strings was kept constant at 0.66 mm (string model BG66 series) to examine the effect of string tension on the acceleration reaction.

To study the effect of string diameters on the acceleration reaction, six badminton racquets were assembled with different diameters of the pull strings, namely 0.62 mm, 0.64 mm, 0.66 mm, 0.68 mm, and 0.70 mm, while the tension of the pull strings was kept constant at 24 lb/ft^2^.

The Yonex Aerosensa (AS series) shuttlecock was selected for the tests as this is a popular choice among badminton players due to its high quality and consistency. This shuttlecock is made of high-quality materials and is designed to provide consistent flying performance. The overall length of a shuttlecock is approximately 2.5 in (63.5 mm), while the diameter of the base is around 0.94 in (24 mm). The weight of a shuttlecock is between 4.74 to 5.50 g, and it consists of 16 feathers attached to a cork base. The Yonex Aerosensa series shuttlecocks are available in different speeds (AS-10, AS-20, AS-30, AS-40, and AS-50) to match different playing conditions and player preferences. The speed of a shuttlecock is determined by the stiffness of its feathers and can range from slow (AS-10) to fast (AS-50). The faster shuttlecocks are suitable for playing in warm conditions and at higher altitudes, while the slower shuttlecocks are better for playing in cooler conditions and at lower altitudes.

**Figure 2:**
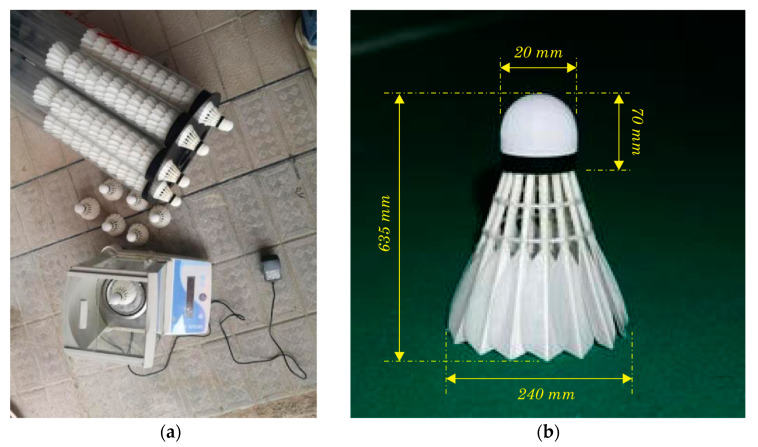
(**a**) Weighing of Aerosensa AS30 shuttlecocks with a precision scale of 0.001 g; (**b**) dimensions of typical shuttlecock.

A digital balance scale (Figure 2a) was used to measure the weight of the badminton racquets, as well as the shuttlecock. Table 1 summarizes the weight of the 10 badminton shuttlecocks used in the tests, which had typical dimensions as shown in Figure 2b.

The 10 shuttlecocks (Aerosensa, AS series) included speeds AS-10, AS-20, AS-30, AS-40, and AS-50. The mean weight values for the shuttlecocks were found to be 5.193, 5.004, 5.119, 5.041, and 4.906 g, respectively, with a variation of less than half a gram between the different speeds. The standard deviation of the mean weight value for each speed was also calculated and found to be 4.8%, 7.2%, 5.1%, 5.7%, and 5.3%, respectively. These results indicate that the weight of the shuttlecocks was consistent and conformed to the regulations set by the Badminton World Federation (BWF). The variation in weight between different speeds of shuttlecocks can affect their flying performance and trajectory, and players often choose shuttlecocks according to their playing conditions and personal preferences. The shuttlecock range offers a variety of speeds to match different playing conditions, allowing players to choose the shuttlecock that best suits their game.

### 2.2. Test Setup

**Figure 3:**
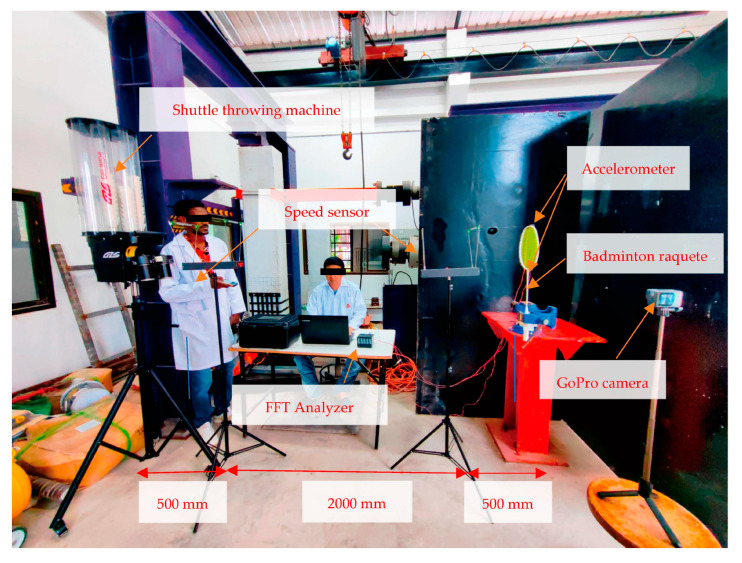
Experimental setup and shuttlecock shooting arrangement.

The experimental setup (see Figure 3) comprised a metal frame measuring 1000 mm in length and 500 mm in width, and it was designed to firmly hold a badminton racquet. The racquet was secured within the metal frame using a manual interlock method, with two stiff clamps holding the racquet in place. The experimental setup included an FFT analyzer, accelerometer, shuttle gun, badminton racquet, and a stand for holding the racquet [4,19]. The procedure for the experimental analysis involved fixing the badminton racquet onto the holding stand and mounting the accelerometer on the frame, which was then connected to the FFT analyzer. To determine the natural frequencies, the frame was impacted at various positions using the shooting machine, as described later.

#### 2.2.1. Accelerometer and Fast Fourier Transformation (FFT) Analyzer

**Figure 4:**
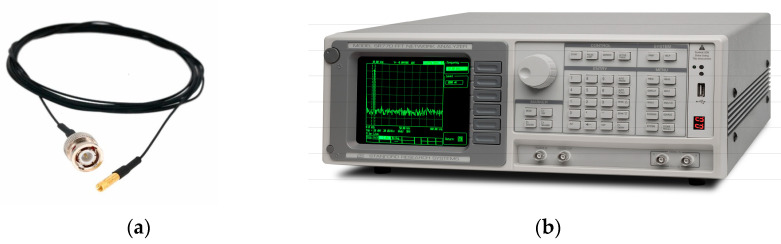
Data acquisition systems: (**a**) accelerometer; (**b**) FFT analyzer.

An accelerometer is a sensor used for measuring acceleration, from which frequency, vibrations, and modes can be obtained [20]. The accelerometer used in this experimental setup (Figure 4a) allowed to determine the overall acceleration and frequency of the badminton racquet. The accelerometer was fixed with glue at the joint where the racquet rod and frame meet. The accelerometer was connected to a fast Fourier transform (FFT) analyzer (Figure 4b). Frequencies were calculated using Dewesoft software version x3 sp9, and a report was generated on the basis of the findings. The FFT spectrum analyzer samples the input signal, computes the magnitude of its sine and cosine components, and eventually displays the spectrum of these measured frequency components [21,22,23].

#### 2.2.2. Shuttlecock Shooting Machine and Speed Measurements

**Figure 5:**
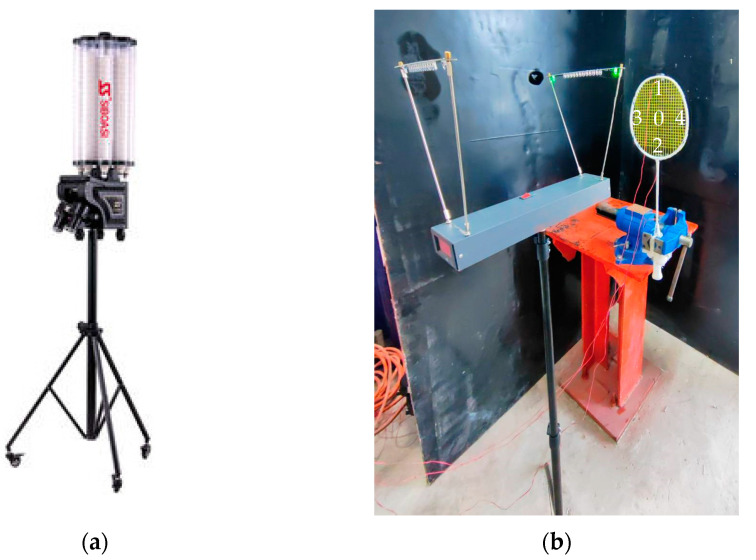
(**a**) Shuttlecock shooting machine; (**b**) speed sensor.

A shuttlecock shooting machine was used to hit the badminton racquets (as depicted in Figure 5a). The shooting machine was positioned at distances of 3 m, 5 m and 8 m from the racquet to allow a parametric analysis. Two positions were considered to measure shuttlecock speeds: position 1 at the exit of the shooting machine, and position 2 at the badminton racquet. Hitting points at the strings were Point #1 at the top, Point #3 on the left, Point #0 at the center, Point #4 at the right, and Point #2 at the bottom. A speed sensor (Figure 5b) measured the speed of the shuttlecocks being shot at the racquet strings. Such sensors are an integral part of onboard systems [24]. A clip of an actual test is included as Supplementary Material.

## 3. Analysis of Test Results

The purpose of this study was to investigate the performance of badminton racquets by examining several variables. The variables considered were the effect of the location of hitting points on the badminton racquet, the effect of the diameter and tension of the strings, and the effect of the string pattern. These variables were analyzed to determine their impact on the performance of the badminton racquet. The study aimed to provide a better understanding of how these variables affect the performance of the racquet, which could lead to improvements in the design and production of badminton racquets. By exploring these variables, the study aimed to contribute to the development of better badminton equipment and enhance the overall playing experience for badminton players. 

**Figure 6:**
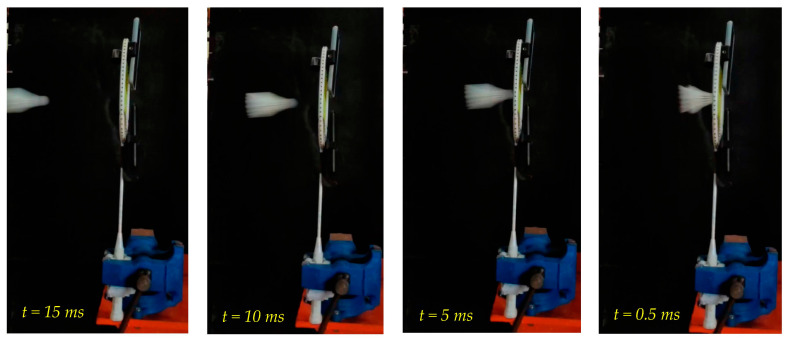
Time lapse of shuttlecock hitting a badminton racquet, 10 ms gap.

The acceleration of the shuttlecock was affected by the tension in the strings. In this study, when the string tension increased from 20 lb to 34 lb, the shuttlecock speed decreased correspondingly. As can be seen in the time lapse in Figure 6, during the impact of the shuttlecock, there was energy transferred from the ball to the strings. When the string tension was too low, a high amount of energy was absorbed, and this led to a low rebound height [18].

### 3.1. Effect of Location of Hitting Points on Badminton Racquet

**Figure 7:**
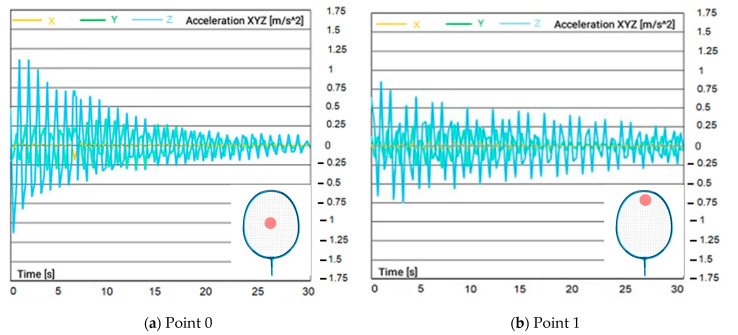

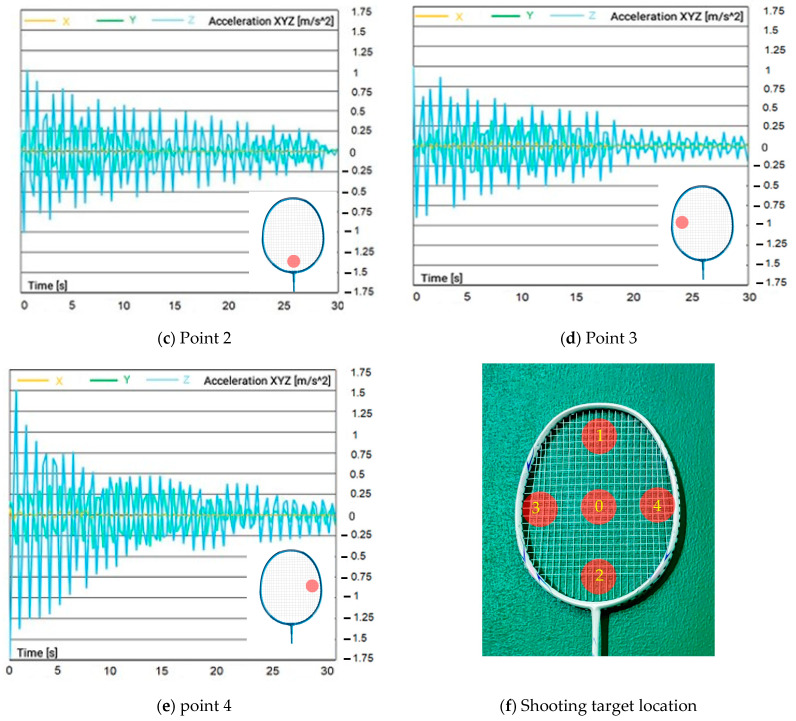
Acceleration recorded at different hitting points: (**a**) center point #0; (**b**) upper top point #1; (**c**) bottom point #2; (**d**) left point #3; (**e**) right point #4; (**f**) shooting locations on the string pattern type A.

The signals recorded from the accelerometer were collected and analyzed to study the behavior of the racquet during hitting. Figure 7a–d show the acceleration vs. time results obtained from the accelerometer. This graph is an essential tool to understand the movement of the badminton racquet during hitting. It shows the variation of acceleration over time and indicates the positions of the racquet at which an acceleration is the highest. This information is crucial to study the dynamics of racquet–shuttle interaction.

The increase in magnitude of acceleration at the center of the badminton racquet, as shown in Figure 7a–d, is a critical finding of this study. This increase in acceleration is attributed to the greater potential energy that the center of the racquet can absorb and release during hitting, compared to other hitting positions. This finding has significant implications for the design of badminton racquets. It suggests that the “sweet spot” of the racquet, which is the point on the racquet where the maximum energy is transferred to the shuttle, is located near the center of the racquet. Understanding the sweet spot is essential for players to hit the shuttle with maximum power and control. Therefore, the findings of this study can be used to optimize the design and performance of badminton racquets for professional and recreational players alike.

### 3.2. Effect of String Diameter and Tension of String

The first variation of the study involved the manipulation of the pull string tension to determine the effect on the coefficient of restitution of the shuttlecock. The results of this variation were recorded and analyzed to determine the effect of the pull string tension on the coefficient of restitution of the shuttlecock. The second variation of the study involved the manipulation of the diameter of the pull strings to determine the effect on the vibration of the shuttle racquet. The six badminton racquets assembled with different diameters of the pull strings were used for this variation. The vibration of the shuttle racquet was recorded and analyzed to determine the effect of the diameter of the pull strings on the vibration of the shuttle racquet.

**Figure 8:**
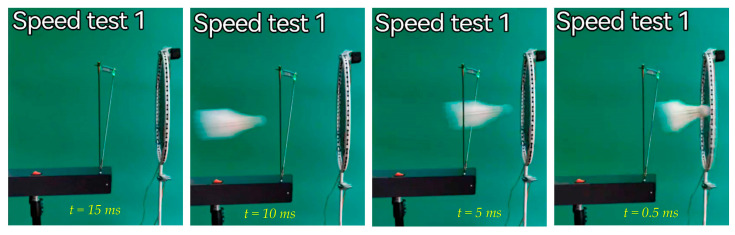
Time lapse of shuttlecock hitting the badminton racquet at the center (position #0).

The speed of the shuttlecock shot at different distances is shown in Table 2. The table shows the results for different distances between the badminton racquet and the shooting machine at two positions: position 1 at the exit of the shooting machine, and position 2 at the badminton racquet. Figure 8 shows a time lapse of shuttlecock hitting the badminton racquet at the center (e.g. Point #0).

#### 3.2.1. Effect of String Diameter

**Figure 9:**
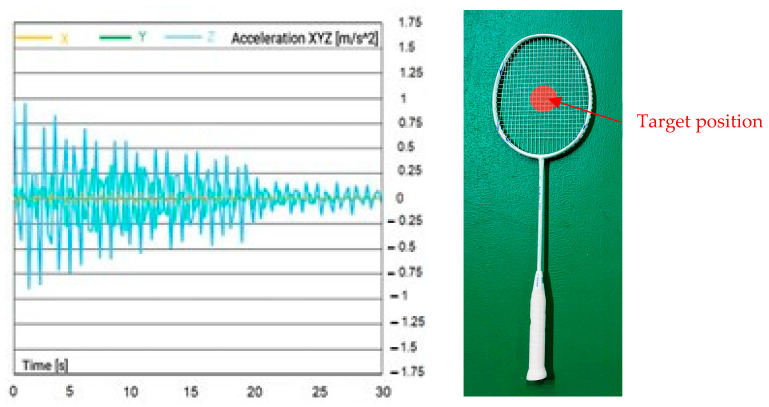
Acceleration vs. time results of badminton racquet with 0.70 mm diameter strings, using the string pattern type A.

**Figure A2:**
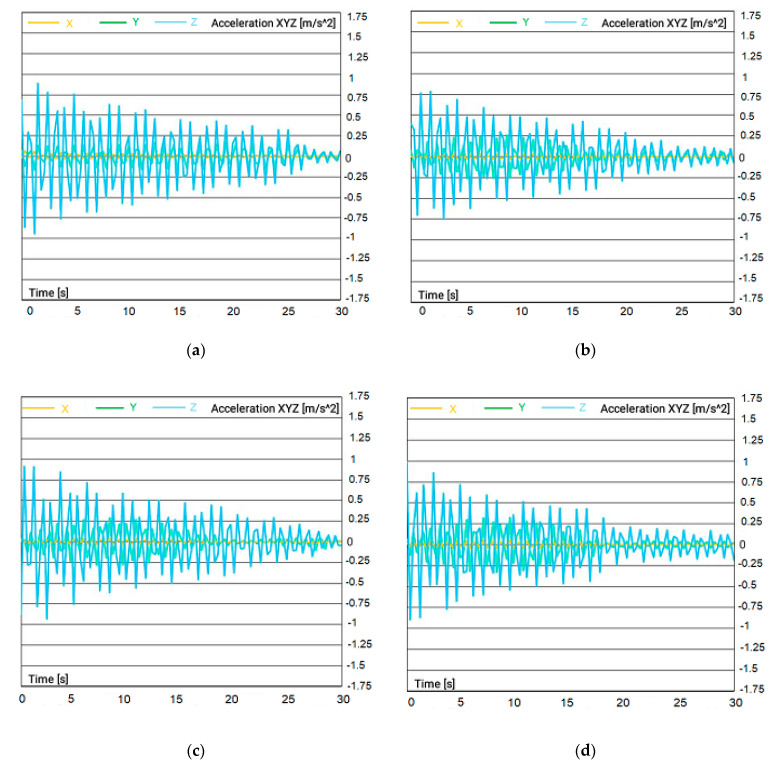
Acceleration time plot of badminton racquet for a diameter of string: 0.62 mm (**a**); 0.64 mm (**b**); 0.66 mm (**c**); 0.68 mm (**d**).

Larger-diameter strings are more durable but less elastic. Conversely, smaller-diameter strings have better elasticity but less endurance and, thus, break more easily [8]. The diameter of the strings on a badminton racquet has a significant impact on the performance of the racquet. When the diameter of the strings is large, the control and elasticity of the shuttlecock improves. This section utilized advanced measurement techniques to explore the vibration response of badminton racquets with varying string diameters. The test results shown in Appendix C report the acceleration vs. time for different string diameters (0.62 mm, 0.64 mm, 0.66 mm, and 0.68 mm). For example, the result in Figure 9 shows that a 0.7 mm string diameter causes the racquet to vibrate in a manner that can lead to decreased accuracy and power. This is attributed to the fact that the racquet is not able to absorb the shock of the shuttlecock impact as effectively as it could with a thicker string diameter. The test results for other diameter strings are shown in Appendix C (Figure A2). In general, stiffer materials were found to produce less vibration in the racquet, leading to improved performance. These findings have important implications for badminton players and racquet manufacturers alike, as they suggest that selecting the right string diameter and material is critical to achieving optimal performance. Overall, the diameter of the strings on a badminton racquet is a crucial factor in determining the performance of the racquet and can make a significant difference in a player’s ability to excel on the court.

#### 3.2.2. Effect of Tension of String

The string tension of their racquets affects their performance on the court. In fact, when the string tension is higher, the player has more control of the shuttlecock. When pulling high strings, the strings are relatively hard, which means that the shuttlecock and racquet face are in contact for a relatively short time. This short contact time allows for greater precision and control over the shuttlecock’s direction and speed, which is crucial for players who want to excel in this sport.

**Figure 10:**
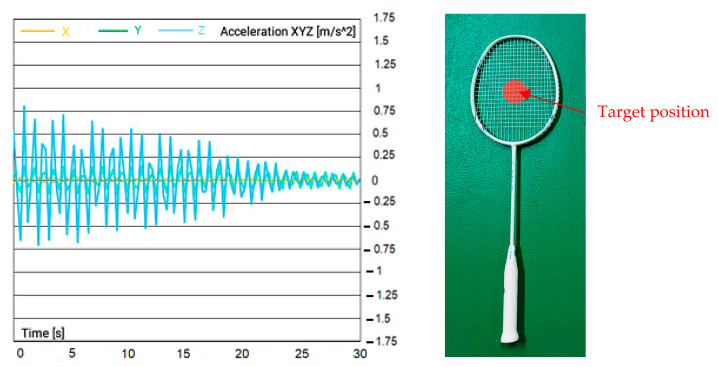
Acceleration–time result of 28 lb string tension using the string pattern type A.

**Figure A3:**
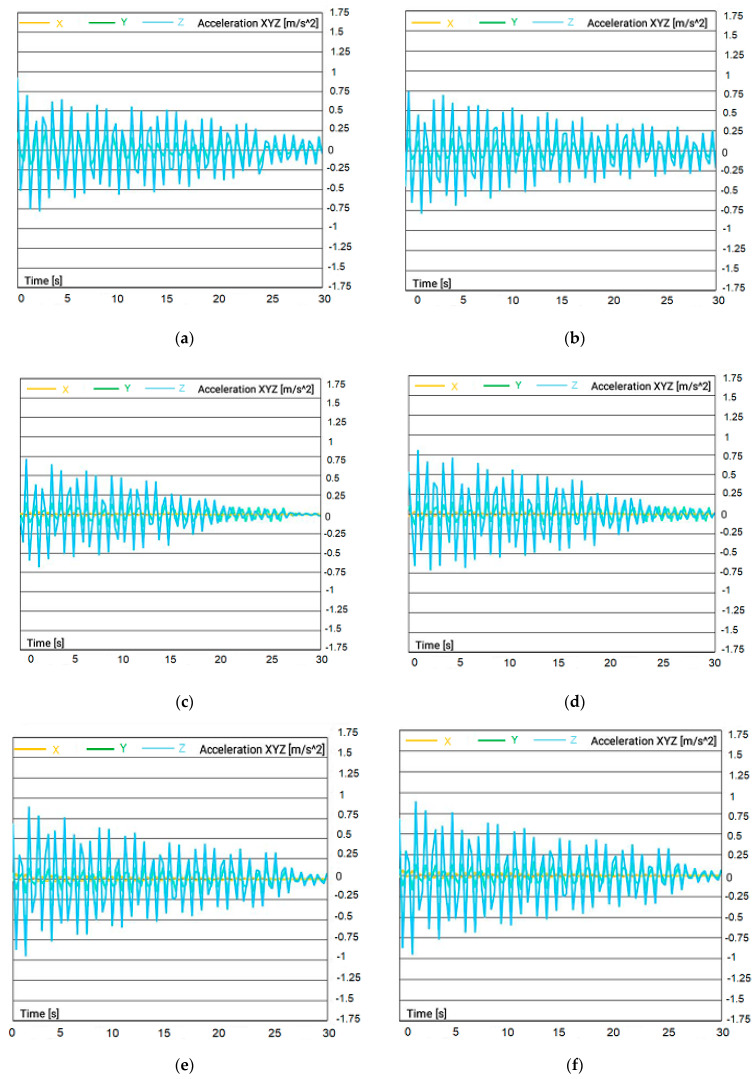
Acceleration time result on the effect of string tension on the performance of badminton racquets: 20 N (**a**); 22 N (**b**); 24 N (**c**); 28 N (**d**); 30 N (**e**); 34 N (**f**).

Figure 10 illustrates the effect of string tension on the performance of badminton racquets for 28 lb tension. The test results for other tension strings are shown in Appendix C, Figure A3. The results show that, for a higher tension of the string, the elasticity of the badminton racquet is lower under impact with the shuttlecock. This means that high string tension is better suited for players who prioritize control over power, while low string tension is better for those who prefer more speed and power in their game. The results also show that the shuttlecock’s speed depends on string tension, with lower tension generating more shuttlecock speed than high tension (20 lb > 22 lb > 24 lb > 28 lb > 30 lb > 34 lb). These results suggest that badminton players should have a good understanding of their individual preferences and modify their strings accordingly. Players can experiment with different string tensions to determine which one is best suited for their playing style and skill level. Some players may prefer a lower string tension for more power and speed, while others may prioritize control and accuracy with higher string tension. In conclusion, understanding the impact of string tension on badminton racquet performance is crucial for players who want to improve their game and reach their full potential on the court.

### 3.3. Effect of String Pattern

**Figure 11:**
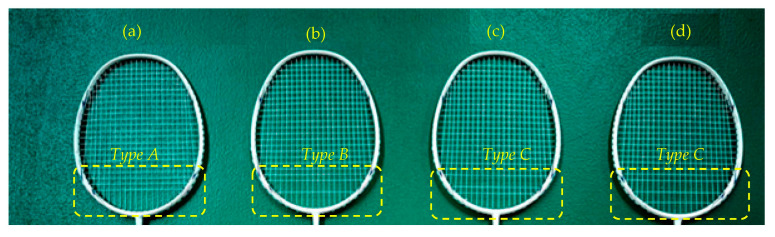
Closeup view of four string patterns examined in the study: (**a**) type A; (**b**) type B; (**c**) type C; (**d**) type D.

One of the critical components of a badminton racquet is the string pattern, which determines how many strings are present and how tightly they are strung. The choice of string pattern can impact a player’s performance and the durability of the racquet. To understand how the string pattern affects badminton racquet performance, a study was conducted to investigate the relationship between string pattern and string breakage. Four similarly sized frames with same strings and tensions but different string arrangement as shown in Figure 11. In this figure, four types of string patterns are shown: A, B, C, and D. The racquet with the more open string pattern or fewer strings will usually have a softer, arm-friendlier response when hit by a shuttlecock. The strings move and rub against each other, causing them to weaken and eventually snap. When the string pattern is more open and the strings have to move more, the frequency of breakage tends to be higher.

**Figure 12:**
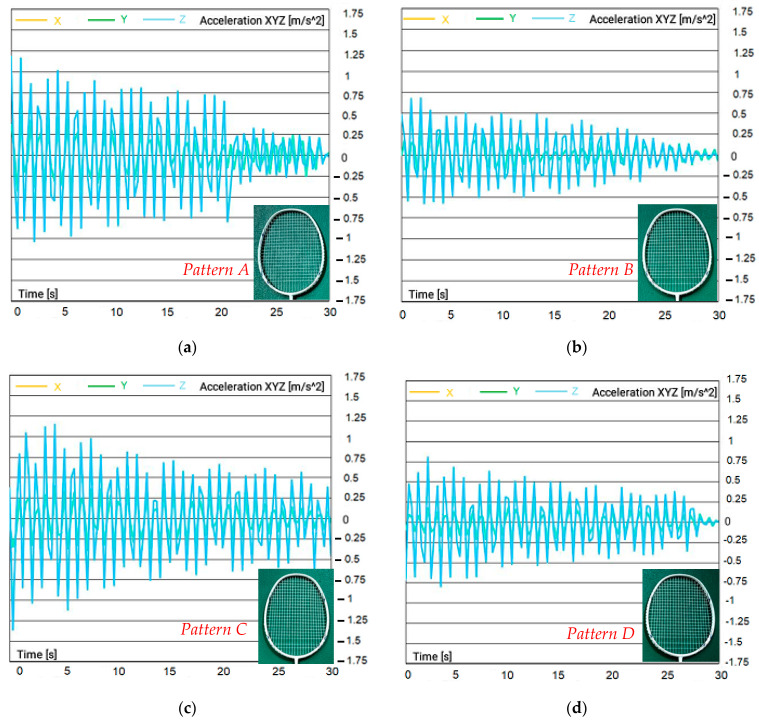
Acceleration vs. time result of badminton racquet for different patterns: (**a**) pattern 1; (**b**) pattern 2; (**c**) pattern 3; (**d**) pattern 4 (shuttlecock hitting at center of racquet point #0).

The results show that, the more open the string pattern, the more the racquet string moved, thus resulting in a higher frequency of breakage. This means that racquets with more open string patterns experience more stress on individual strings during impact, leading to an increased likelihood of breakage. Figure 12 illustrates the acceleration vs. time results for different string patterns measured at the center of the racquet (point #0). The results show that the badminton racquet with a string pattern type C had a higher vibration response than other racquets when the shuttlecock hit. This finding suggests that racquets with this string pattern experienced more stress and strain during impact, which could lead to reduced durability and increased chances of breakage. This in turn highlights the importance of considering different string patterns when selecting a badminton racquet. 

Overall, the choice of string pattern is an essential factor to consider when selecting a badminton racquet. Players must balance the need for a high-performance racquet with the desire for durability and longevity. By understanding how string pattern affects a racquet’s performance and durability, players can select a racquet that best suits their needs and playing style. The results of this study provide valuable insights for both players and manufacturers looking to optimize badminton racquet design and performance.

### 3.4. Effect of Distance between Cock and Badminton Racquet

**Figure 13:**
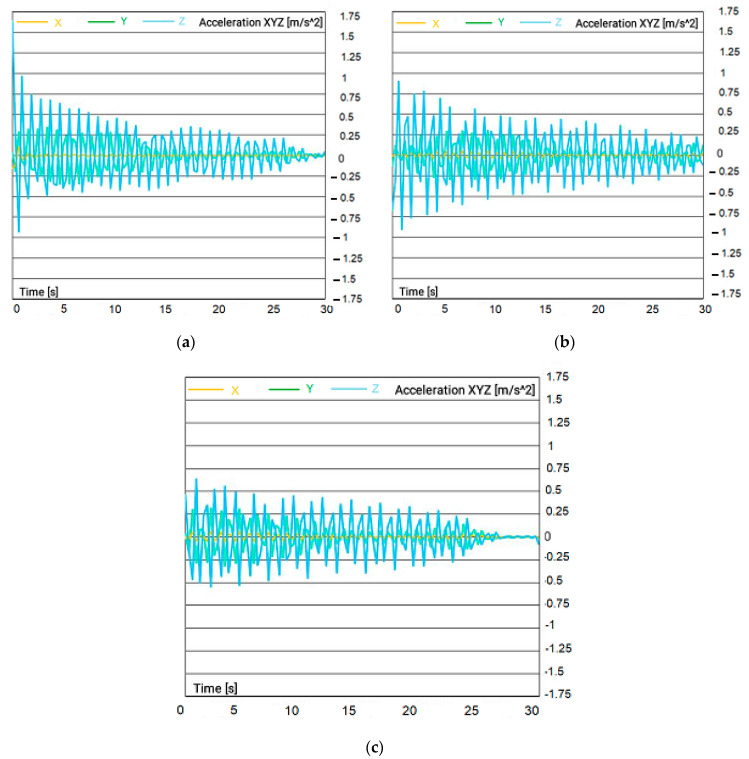
Acceleration vs. time result of badminton racquet for three distances: (**a**) 3 m; (**b**) 5 m; (**c**) 8 m.

This section considers the effect of the distance between the shooting machine and the racquet on the performance of racquets themselves. Figure 13 shows the acceleration results of the tests shooting the shuttlecock at distances of 3, 5, and 8 m from the racquet. The results revealed that the selection of the right racquet can have a significant impact on the player’s ability to hit the shuttlecock accurately at different distances. Therefore, badminton players must select the appropriate racquet for their preferred distance range.

Additionally, understanding the impact of string tension and other factors on racquet performance can help players make informed decisions when choosing a racquet. For instance, if a player prefers to play from a distance, they may choose a racquet with a higher string tension to maximize control and accuracy. Alternatively, if a player prefers speed and power, they may opt for a racquet with lower string tension. This highlights the importance of understanding the mechanics of the game and the impact of different factors on racquet performance. In conclusion, selecting the right badminton racquet is crucial for a player’s performance on the court. To optimize performance, factors such as string pattern, hitting point, string diameter, and tension must be considered. Moreover, the impact of distance on racquet performance should not be ignored, and players should select a racquet that is best suited for their preferred distance range.

### 3.5. Tradeoff between Power and Control with String Tension

Table 3 summarizes the tension of strings in racquet, speed of shuttlecock, and power obtained from the tests. The results in the table show that lower string tension leads to a greater rebound speed, consistent with the observed phenomenon of increased string deformation upon impact, resulting in enhanced energy storage and faster rebound. Conversely, higher string tension offers improved control due to stiffer strings, ensuring a consistent and predictable response. Adjusting string tension entails a tradeoff between power and control. As shown in Table 3, increasing tension from 20 lb to 34 lb enhanced power by 32% through greater energy transfer to the shuttlecock. Conversely, lower tension enhanced control by facilitating greater precision and accuracy. However, lower tension may decrease power due to reduced efficiency in energy transfer caused by increased string deformation.

### 3.6. Main Observations for Analysis

The parametric study in Section 3 shows the mechanics of the racquet and the impact of different factors on its performance. The sweet spot, located at the center of the badminton racquet, is where maximum energy is transferred to the shuttlecock during a hit. This results in an increase in acceleration magnitude, making it crucial for players to aim for this sweet spot to achieve the desired shot. However, the choice of string diameter can significantly impact the performance of the racquet. A 0.7 mm string diameter can cause the racquet to vibrate, reducing accuracy and power. Hence, players must choose a string diameter that is appropriate for their playing style and preferences. String tension is another critical factor that affects the performance of the racquet. Higher string tension, such as 34 lb, results in lower elasticity of the racquet under impact, making it ideal for control-focused players. On the other hand, lower string tensions generate more shuttlecock speed, making it better for players who prefer speed and power. The string pattern also plays a crucial role in the performance of the racquet. Badminton racquets with a string pattern such as type C (see Figure 11) have higher vibration during hitting, which can lead to reduced durability and increased chances of breakage.

On the basis of test results presented in the previous sections, a 0.7 mm string diameter and 34 lb string tension at the center (point #0) were selected for further numerical and analytical studies. The objective was to determine the best string type, deformation, and maximum stress generated during the impact between the shuttlecock and the badminton racquet.

## 4. Numerical Simulations for Impact Analysis

The main objective for the finite element (FE) model was to capture the behavior of the string when the ball of the shuttlecock impacts at different locations. To ensure that the model accurately replicated the motions observed in the images obtained during the tests, the lowest nodes of the handle were designated as rigid and connected to an additional node positioned in the middle. This configuration allowed the model to capture all types of motion, including translations and rotations. Although this approach may create localized stress concentrations around the rigid nodes, it does not impact the desired results since the simulations focused solely on the elastic response, with the stress distribution falling outside the scope of analysis.

### 4.1. FE Geometry, Meshing, and Material Properties

The von Mises stress distribution was assessed using the finite element method and ANSYS2022 R2® software (version 22.2). The lengths of the badminton racquet strings were determined according to standard specifications. The racquet head measured 680 mm in length and 230 mm in width. The diameter of the racquet strings was set at 0.7 mm. The material properties of the strings, which were made of nylon and natural gut, included a Young’s modulus of 7200 MPa, a Poisson’s ratio of 0.3, and a density of 1100 kg/m^3^ for nylon, and a Young’s modulus of 5700 MPa, a Poisson’s ratio of 0.27, and a density of 1320 kg/m^3^ for natural gut [11]. The stringed area adhered to regulations defined by the Badminton World Federation, with a length of 280 mm and a width of 220 mm [25]. Frictional contact, employing a friction coefficient of 0.1, was considered between the strings and between the ball and strings [11]. Both ends of each string were assumed to be fixed, and the simulation utilized an implicit solver. Modal analysis was conducted on the clamped racquet to evaluate its vibration modes. Due to its slim design, the shaft of the racquet deflects when accelerated, resulting in a spring effect during the stroke, which could lead to higher shuttlecock speeds. Therefore, it was of interest to examine the deflection behavior of the racquet to understand how it affected the shuttlecock for different strings. The deflection analysis required a full swing at each string location during impact to analyze the deformation of the racquet. Thus, for each parameter, a total simulation of the stroke was needed. The approach to obtain the deflections was to compare the deformed racquet against the still model.

**Figure 14:**
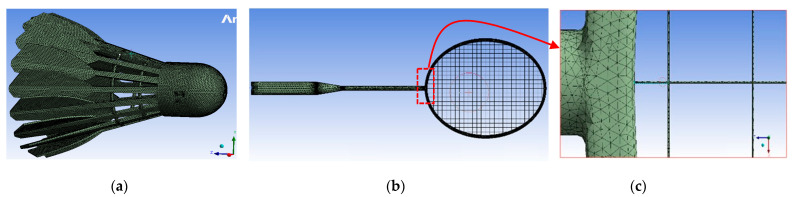
Meshing in ANSYS: (**a**) shuttlecock; (**b**) badminton racquet; (**c**) close up view of T-zone skeleton part.

A mesh sensitivity test was conducted to determine the element size, considering the diameter of the string. Accordingly, a mesh optimum size of 0.5 mm was chosen as shown in Figure 14a–c. The number of nodes and number of elements in the model were 79,368 and 160,748, respectively. The natural frequency of the different modes was calculated using modal analysis in ANSYS, whereas the impact analysis was conducted using explicit dynamics in ANSYS AUTODYNE to find the optimal material. The greatest strength and toughness to resist fracture were determined by studying the impact force.

### 4.2. Loading and Boundary Conditions

In a laboratory test to analyze the behavior of a fixed badminton racquet and shuttlecock under impact, loading and boundary conditions are essential to ensure accurate results. The badminton racquet was fixed at the bottom, which meant that it was securely mounted or clamped to prevent any movement during the test. This condition simulated the grip of the player and ensured that the racquet remains stationary throughout the experiment. The shuttlecock, on the other hand, was subjected to a continuous and controlled loading condition. The shuttlecock shooting machine shot the shuttlecock toward the fixed racquet at a speed of 94 m/s. Throughout the experiment, the boundary conditions are closely monitored to ensure that they remain constant. The lab environment, including temperature, humidity, and air pressure, was controlled and kept constant throughout the test. This ensured that the experiment’s variables were minimized, and the results were reliable and accurate. When the shuttlecock hits the fixed badminton racquet, several forces come into play, such as impact, friction, and deformation. These forces cause stress on the racquet and shuttlecock, resulting in deformation and vibration. By analyzing the loading and boundary conditions and the forces generated during the experiment, researchers can gain valuable insights into the behavior of the badminton racquet and shuttlecock under impact. This information can be used to develop better badminton equipment, improve player performance, and enhance overall game experience.

### 4.3. Analysis Procedure

**Figure 15:**
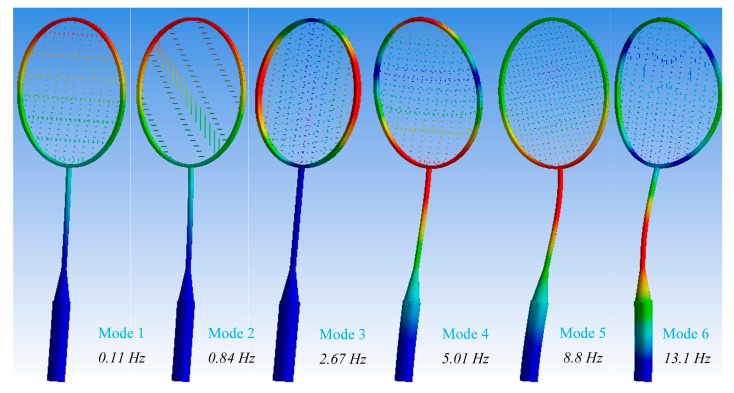
Natural frequency of badminton racquet with synthetic gut string material.

**Figure A4:**
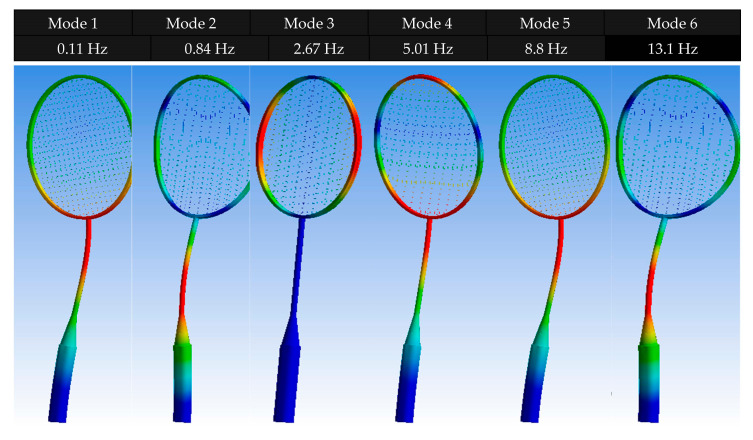
Natural frequency of badminton racquet for synthetic gut string material.

**Figure A5:**
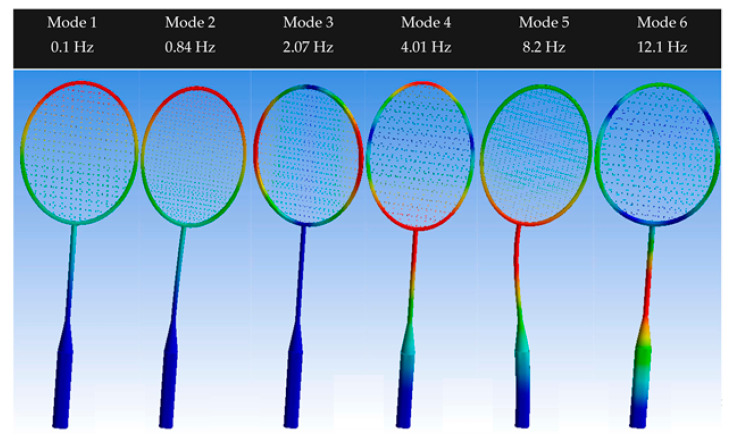
Natural frequency of badminton racquet for natural gut string material.

**Figure A6:**
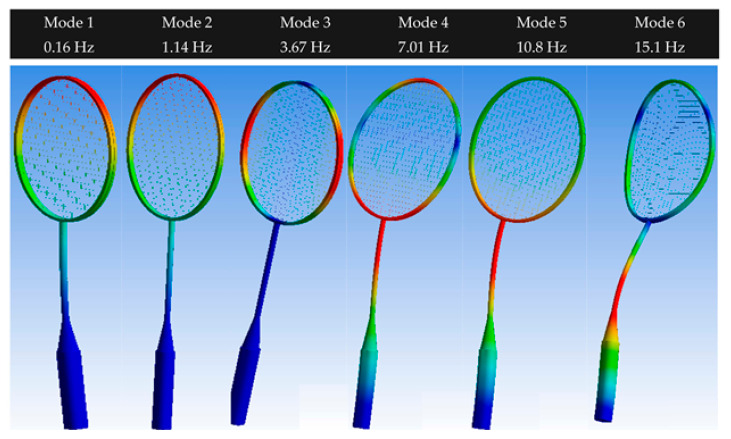
Natural frequency of badminton racquet for Kevlar string material.

**Figure A7:**
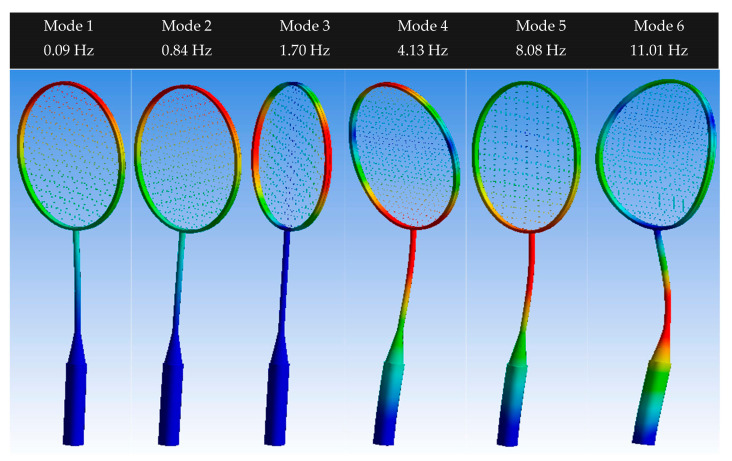
Natural frequency of badminton racquet for polyester string material.

Upon impact with the strings, the tension in the local area surrounding the point of impact can significantly increase, reaching several hundred pounds. This results in the deformation of the racquet and the frame snapping back, overshooting its original configuration, and subsequently oscillating for a period, the duration of which depends on the damping applied. Therefore, a finite element model of a racquet (excluding the rubber grip) was utilized to simulate the free vibrational mode and examine its sensitivity to the boundary conditions imposed by the grip. Figure 15 and in Appendix D (Figure A4, Figure A5, Figure A6 and Figure A7) show the numerical result of the natural frequencies of different modes for badminton racquets with different string materials, which are summarized in Table 4.

## 5. Analytical Calculations

### 5.1. Analysis of Natural Frequency

A precise evaluation of the natural frequencies of systems in the vibration analysis is a critical issue in various applications to eliminate resonance. To this aim, this section gives a detailed description of the analysis of racquet [26]. The analysis was carried out by applying Euler–Bernoulli’s theory, thus deriving the necessary formulations as the dimensions of the racquet meet the criterion of thin column theory [27,28]. In the analytical case, a model was characterized by a partial differential equation with respect to position and time coordinates. On the basis of the equation formulation (shown in Appendix A), the natural frequency $f_{n}$ can be calculated as a function of the circular frequency $\omega_{n}$ using Equation (1).

(1) $$f_{n}=\frac{\omega_{n}}{2\pi }.$$

Table 5 presents the natural frequencies of different modes of four types of strings commonly used in badminton racquets: nylon string, natural gut string, Kevlar string, and polyester string. The natural frequency of a string is a measure of its stiffness and elasticity, and it determines the behavior of the string when it is subjected to various dynamic loads during the game. The evaluation of the natural frequency of strings is an essential aspect of racquet design, as it can have a significant impact on the performance and feel of the racquet.

The results presented in Table 5 show that the badminton racquet with Kevlar string had a better elastic performance during impact compared to the other three types of strings. This is because Kevlar is a high-strength synthetic fiber with excellent toughness and resistance to impact, making it an ideal material for use in badminton racquet strings. The natural frequency of the Kevlar string is higher than that of the other strings, indicating that it is stiffer and more elastic, which enhances the performance of the racquet. The elastic performance of a badminton racquet is a crucial factor that determines its power, control, and feel. A racquet with high elastic performance can transfer more energy from the player’s swing to the shuttlecock, resulting in a more powerful shot. It can also provide better control and accuracy, allowing the player to place the shuttlecock precisely in the opponent’s court. Moreover, a racquet with good elastic performance can provide a better feel, allowing the player to sense the shuttlecock’s position and speed more accurately, which is essential for making quick and precise shots.

In conclusion, the natural frequency of badminton racquet strings is a critical factor in racquet design and performance. A racquet with high elastic performance can provide more power, control, and feel, which are essential for success in badminton. Therefore, the evaluation of the natural frequency of strings is an essential aspect of racquet design, and the use of high-performance materials such as Kevlar can enhance the performance of the racquet.

### 5.2. Impact Analysis

**Figure 16:**
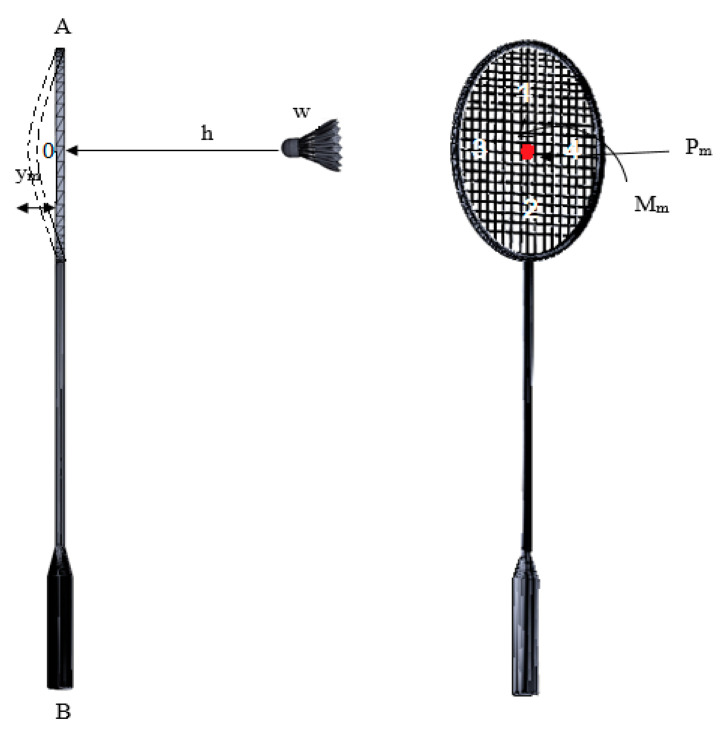
Racquet–shuttlecock impact locations.

The bending moment and stresses experienced by the badminton racquet due to impacts are studied in this section [29,30]. In badminton, the strings of the racquet are subjected to significant dynamic loads during play, including impacts with the shuttlecock. These loads can cause deformation and stress in the strings, which can affect the performance and durability of the racquet [31,32,33,34,35,36,37]. Therefore, it is essential to study the behavior of the strings under these dynamic loads to optimize their design and enhance the performance of the racquet. In this section, analytical analysis was used to evaluate the deformation and stress in nylon strings and other materials under collision with a shuttlecock at the center. Likewise, the maximum deformation and stress of the racquet were also studied. The mean weight of the shuttlecock for impact analysis was taken from Table 1 as 4.9 g from a horizontal distance of 5 m with a speed of 100 m/s (recorded from the test), as shown in Figure 16. The force exerted by the shuttlecock can be calculated using Equation (2).

(2) $$w=m\times g=4.9\;g\times 9.81\frac{m}{s}=48.07\times 10^{-3}N,$$

where m is the mass, and g is the acceleration due to gravity.

If a shuttlecock is shot to the racquet AB and hits at point #0, then the maximum deflection can be written as follows: work of moving weight = $w\left(h+y_{m}\right)$, and energy = $\frac{1}{2}m\times v^{2}$, where the variables are defined in Figure 16. As a result, the strain energy can be calculated as the sum of weight and energy. The maximum deflection (Equation (3)) and stress (Equation (4)) are calculated as follows using the equation formulation and variables defined in Appendix B:

(3) $$y_{m}=y_{st}\left\{1+\sqrt{1+\frac{2h}{y_{st}}+\frac{mv^{2}}{ky_{st}}}\right\},$$

(4) $$\sigma_{m}=\frac{3Ey_{m}r_{o}}{L_{ab}^{2}}.$$

The results of the analysis in Table 6 show that, for nylon strings, the maximum deformation and the corresponding maximum stress were 2.1 mm and 45.13 MPa, respectively, when the shuttlecock hits at the central Point #0. The maximum deformation and maximum stress were also determined for other materials under the same loading conditions. The analysis revealed that different materials exhibit different deformation and stress behavior, with some materials exhibiting better elastic properties and higher resistance to deformation and stress. In conclusion, the behavior of badminton racquet strings under dynamic loads is crucial for optimizing the design and performance of the racquet. The results in this section showed that different materials exhibit different deformation and stress behaviors under collision at the center. These findings can inform the design of badminton racquet strings to enhance their performance and durability.

### 5.3. Compariosn of Predicted Natural Frequencies vs. Analytical Values

**Figure 17:**
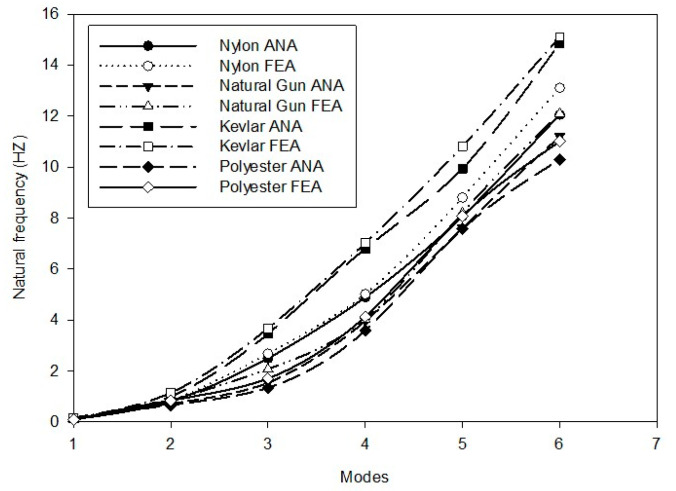
Comparison of frequencies calculated by FE and analysis of badminton racquets with different string materials.

Figure 17 compares the natural frequency of a string calculated using both FE analysis and analytical methods for various materials. The finite element analysis is a numerical technique used to solve complex engineering problems by dividing the system into smaller elements that can be easily analyzed. On the other hand, the analytical method involves solving mathematical equations on the basis of the properties of the materials and the geometry of the system. In this study, both methods were used to calculate the natural frequency of a string made of different materials. The comparison between the two methods showed that the frequencies calculated by both methods were in good agreement, with a difference of only 5%. This indicates that the FE analysis is a reliable method for predicting the natural frequency of strings for different materials. Furthermore, the good agreement between the two methods highlights the accuracy and effectiveness of the FE method for solving complex mechanical problems. 

To investigate the badminton racquet with different string types subject to impact loading, different hitting positions and types of strings were used as key variables. During the collision between the shuttlecock with the racquet strings, the impact force acting upon the racquet with natural gut string material was 2.4%, 19.4%, and 25.1% at the center (point #0), 0.8%, 21.3%, and 7.1% at point #1, 3.1%, 10.62%, and 6.9% at point #2, 2.2%, 1.9%, and 3.5% at point #3, 1.74%, and 1.84%, and 0.3% at point #4, higher than the racquets with Kevlar, nylon, and polyester, respectively. The material of the string plays a significant role in this collision, and the study of its properties is crucial to improving the player’s performance. The results of a recent study revealed the percentage of natural gut string material that is present at different points during collision.

At the center point (point #0), the natural gut string material was found to be the highest string tension (stress value), with a percentage of 2.4%. This indicates that the string material at the center of the racquet is more elastic, allowing for better energy transfer during the collision. At point #1, the percentage of natural gut string material was found to be 0.8%, which was lower than the center point. However, it was still higher than the other string materials, indicating that, even at the point of impact, the natural gut string material was superior. Moving on to point #2, the percentage of natural gut string material increased to 3.1%, which was higher than at the center point. This increase in natural gut string material suggests that the material was more concentrated toward the outer edges of the racquet string. At point #3, the percentage of natural gut string material was found to be 2.2% thus indicating that the material was still present (albeit in lower concentrations) at the outermost points of the racquet string. Lastly, at point #4, the natural gut string material was found to be 1.74%, which was still higher than the other string materials. This indicates that, even at the highest impact point, the natural gut string material was superior in terms of impact strength than Kevlar, nylon, and polyester.

The results indicate that the natural gut string material is more elastic and provides better energy transfer during the collision. Additionally, the study also provides valuable insights into the concentration of the natural gut string material at different points during the collision. These findings can be utilized by players and manufacturers to improve the design and performance of the racquet strings, ultimately leading to better performance on the badminton court.

This study investigated the collision of a shuttlecock with a racquet string made of Kevlar material. The Kevlar string exhibited varying degrees of deformation and energy transfer at different points on the string bed. The results show that the energy transfer during the collision was highest at the center of the string bed, with values of 24.8%, 47.4%, and 56.7% for different impact speeds. This suggests that the center of the string bed is the most effective area for generating power and speed in badminton strokes.

The study also found that the energy transfer at point #1 on the string bed was lower compared to the center, with values of 9.2%, 27.9%, and 37.1% for different impact speeds. This is likely due to the lower tension and stiffness of the string at this point, resulting in less energy transfer. At point #2, located closer to the frame of the racquet, the energy transfer was higher, with values of 17.1%, 63%, and 70% for different impact speeds. This suggests that the strings in this area have higher tension and stiffness, making them more effective in transferring energy to the shuttlecock. Moreover, the study found that the energy transfers at points #3 and #4 on the string bed were also significant, with values ranging from 7.9% to 49.3% and 35.7% to 51.8%, respectively. These results suggest that players can use different points on the string bed to achieve different shot trajectories and speeds, depending on their playing style and the situation on the court. Understanding the energy transfer characteristics of the Kevlar string at different points on the string bed can help players optimize their stroke mechanics and improve their performance on the court.

### 5.4. Comfort Tests with Actual Players

**Figure 18:**
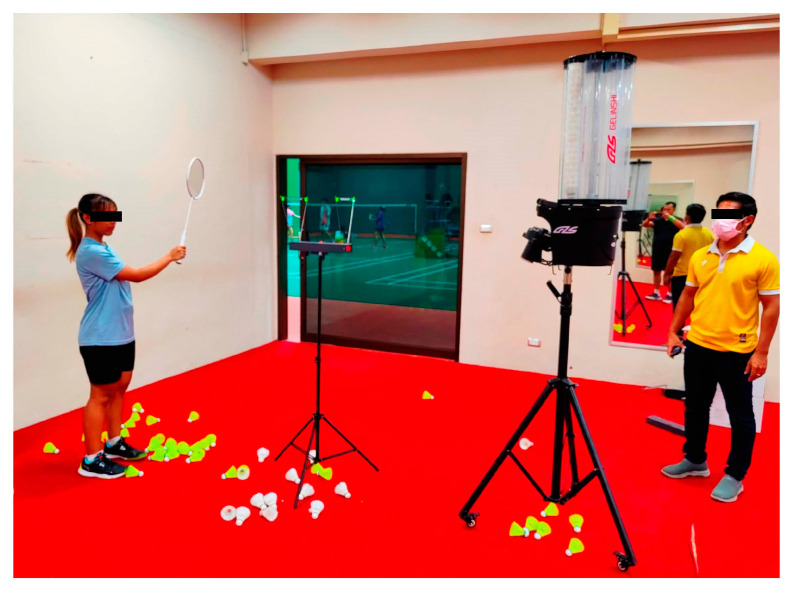
Effect of different string pattern, tension, diameter, and hitting reaction on a professional player (photo taken at Badminton Sport Center, Walailak University).

A final investigation shed light on the effect of shuttlecock impact on players’ comfort and safety, as well as badminton racquet performance. To achieve this, shuttlecocks were shot to racquets held by real players, as shown in Figure 18. The study found that the center of the badminton racquet experienced a greater magnitude of acceleration during hitting compared to other positions. This suggests that the sweet spot of the racquet, where the maximum energy is transferred to the shuttlecock, is located near the center of the racquet. By optimizing the design and performance of badminton racquets on the basis of these findings, players can hit the shuttle with maximum power and control while maintaining comfort and safety. Additionally, the study revealed that using a 0.7 mm diameter string can decrease accuracy and power due to increased vibration, while higher string tension can decrease the elasticity of the racquet upon impact. Players who prioritize control over power may prefer higher string tension, while those who prefer more speed and power may opt for lower tension. Furthermore, the study highlights the importance of considering string pattern when selecting a badminton racquet, as certain patterns may experience more stress and strain during impact, potentially leading to reduced durability and increased chances of breakage.

It should be noted that, whilst this study examined the materials, tension, diameter, and patterns of strings, other factors influence the manufacturing process of badminton racquets. However, the basic conclusions drawn in this study can assist manufacturers in selecting the most suitable string material depending on a desired racquet performance, thus potentially reducing production costs. It is suggested that manufacturers perform more comprehensive experimental and numerical analysis of different string materials available in the market so that evidence-informed decisions can be taken to balance quality and affordability, optimization of production processes, and minimization of procurement costs. Manufacturers can also fine-tune string tensions and string diameters to improve speed and power while considering economic factors.

## 6. Conclusions

This study experimentally, numerically, and analytically investigated the performance of different string materials (Kevlar, synthetic gut, natural gut, and polyester) on badminton racquets based on vibration and impact tests with a shuttlecock. Racquets were hit at different points using a shooting machine. Finite element (FE) analyses were also performed to further analyze the dynamic behavior of badminton racquets with different string materials. On the basis of the results presented in this study, the following conclusions can be drawn:

- The natural frequency of a racquet with Kevlar strings was significantly higher than that of racquets with synthetic gut, natural gut, or polyester string materials. Specifically, the natural frequency of a racquet made of carbon graphite and epoxy resin was 23.0%, 30.7%, and 36.2% higher than that of racquets with synthetic gut, natural gut, and polyester string material, respectively.
- The change in string tension can have a significant impact on shuttlecock speed, with an increase or decrease of up to an average of 10%. This factor can influence achievement and potentially provide an advantage to athletes during a competition. The study found that increasing the string tension could decrease the elasticity of the string by up to an average of 15%. Similarly, increasing the string diameter also decreased the elasticity of the string by up to an average of 20%.
- The center of the racquet, being the point of maximum impact, accounts for up to an average of 57% of the total impact force. Additionally, the translational and rotational contributions to shuttlecock speed can be retained, providing some information about player strength and skill.
- Overall, understanding the effects of different factors on racquet and shuttlecock performance can help players and coaches make more informed decisions and improve their performance on the playing court. The experimental and FE results in this study suggest that ad hoc tuning of string tension and string diameter can potentially lead to better racquet performance. Moreover, measuring the kinematics of the racquet can provide further insight into both player and racquet’s performance.

## Figures and Tables

**Table 1:** Weight of badminton shuttlecocks (units: g).

Sample No.	Yonex Shuttlecock Aerosensa Series				
	AS-10	AS-20	AS-30	AS-40	AS-50
1	5.159	5.062	5.102	5.049	4.860
2	5.185	5.013	5.156	5.057	4.892
3	5.255	4.916	5.137	4.937	5.021
4	5.249	5.141	5.144	5.008	4.971
5	5.146	5.030	5.181	5.006	4.888
6	5.245	4.991	5.021	5.054	4.878
7	5.124	4.954	5.120	4.965	4.927
8	5.131	4.889	5.036	5.114	4.918
9	5.208	5.066	5.125	5.118	4.869
10	5.190	4.973	5.170	5.092	4.832
Average	5.193	5.004	5.119	5.041	4.906
SD.	0.048	0.072	0.051	0.057	0.053

**Table 2:** Speed of shuttlecock at three different distances from the racquet setup (units: m/s).

3 m		5 m		8 m	
Position 1	Position 2	Position 1	Position 2	Position 1	Position 2
93.4 m/s	91.4 m/s	93.1 m/s	87.4 m/s	92.9 m/s	82.8 m/s

**Table 3:** Results from tension of strings, speed of shuttlecock and power.

Tension per Single String (lb)	Tension (N)	Speed (m/s)	Power (kJ/s)
20	89	91.4	8.14
22	97.9	87.1	8.52
24	106.8	86.0	9.19
28	124.6	84.1	10.47
30	133.5	82.5	11.02
34	151.3	79.2	11.95

**Table 4:** Numerical result of frequency of badminton racquet with different string materials.

String Material	Mode 1	Mode 2	Mode 3	Mode 4	Mode 5	Mode 6
Synthetic gut	0.11 Hz	0.84 Hz	2.67 Hz	5.01 Hz	8.8 Hz	13.1 Hz
Natural gut	0.1 Hz	0.84 Hz	2.07 Hz	4.01 Hz	8.2 Hz	12.1 Hz
Kevlar	0.16 Hz	1.14 Hz	3.67 Hz	7.01 Hz	10.8 Hz	15.1 Hz
Polyester	0.09 Hz	0.84 Hz	1.70 Hz	4.13 Hz	8.08 Hz	11.01 Hz

**Table 5:** Analytical result of natural frequency of badminton racquet for different string materials.

$f_{n}$ (Hz)	Type of String Material			
	Nylon	Natural Gut	Kevlar	Polyester
$f_{1}$	0.1	0.09	0.13	0.083
$f_{2}$	0.81	0.71	0.99	0.658
$f_{3}$	2.49	1.53	3.45	1.334
$f_{4}$	4.88	3.95	6.79	3.575
$f_{5}$	8.06	7.6	9.93	7.563
$f_{6}$	12.05	11.2	14.84	10.3

Note: *f_n_* = natural frequency at nth mode in Hz.

**Table 6:** Analytical result of maximum stress and deformation of badminton racquet for different string materials.

Impact Points	Nylon String		Natural Gut String		Kevlar String		Polyester String	
	δ_max_ (mm)	σ_max_ (MPa)	δ_max_ (mm)	σ_max_ (MPa)	δ_max_ (mm)	σ_max_ (MPa)	δ_max_ (mm)	σ_max_ (MPa)
0	5.8	451.3	8.30	560.0	4.36	546.35	10.1	419.3
1	2.7	417.2	3.40	530.0	2.45	526.44	3.9	492.7
2	2.1	439.0	4.71	491.2	1.74	476.6	5.8	456.9
3	2.5	447.0	3.03	457.4	2.08	447.03	4.1	441.6
4	2.2	453.3	3.15	461.8	2.03	453.74	4.2	460.9

**Figure A1:**
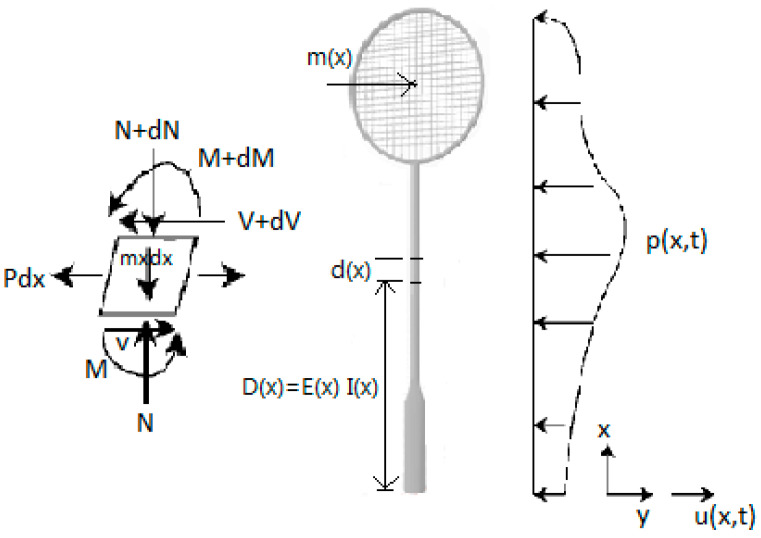
Force distribution and vibration response of racquet during collision with cock.

## Data Availability

Not applicable.

## Supplementary Materials

Accelerometer, Fast Fourier Transformation (FFT) Analyzer, Shuttlecock shooting machine, badminton racket, shuttlecock and Speed measurements. A short clip recorded during testing can be found at the following link: https://youtu.be/9qZtXbV6nSc.

## Appendix A. Equation Derivation of Natural Frequency

The governing equation for vibration of slab can be written as $\sum F_{v}=0$.

$$\frac{\partial V\left(x\right)}{\partial x}=P\left(x,t\right)-m\left(x\right)\frac{\partial^{2}u\left(x,t\right)}{\partial t^{2}}.$$

(A1) $$m\left(x\right)\frac{\partial^{2}u\left(x,t\right)}{\partial t^{2}}+\frac{\partial V\left(x\right)}{\partial x}=P\left(x,t\right).$$

From the diagram above, the transversal shear and bending moment can be expressed as follows:

(A2) $$V\;\left(x,t\right)=\frac{\partial M\left(x\right)}{\partial x},M\;\left(x,t\right)=D\left(x\right)\frac{\partial^{2}u\left(x,t\right)}{\partial t^{2}}\;,\mathrm{and}\;m\left(x\right)=\rho \left(x\right)A\left(x\right).$$

Substituting Equation (A2) into Equation (A1),

$$\rho \left(x\right)A\left(x\right)\frac{\partial^{2}u\left(x,t\right)}{\partial t^{2}}+\frac{\partial^{2}}{\partial t^{2}}\left[D\left(x\right)\frac{\partial^{2}u\left(x,t\right)}{\partial t^{2}}\right]=P\left(x,t\right),$$

(A3) $$\rho A\frac{\partial^{2}u\left(x,t\right)}{\partial t^{2}}+EI\frac{\partial^{4}u\left(x,t\right)}{\partial t^{4}}=0.$$

The above is the general governing differential equation for non-prismatic Bernoulli theory without considering damping.

According with Euler’s Bernoulli theory, the response of the system can be obtained via the separation method using Equation (A4).

*U* (*x*,*t*) = ϕ(*x*) × *q*(*t*). (A4)

Therefore Equation (A3) becomes

$$\phi \left(x\right)\frac{\partial^{2}q\left(t\right)}{\partial t^{2}}+\frac{EI}{\rho A}\frac{\partial^{4\;\phi \left(x\right)}}{\partial t^{4}}q\left(t\right),\frac{EI}{\rho A}\left(\frac{1}{\phi \left(x\right)}\right)\frac{\partial^{4\;\phi \left(x\right)}}{\partial t^{4}}\;=\;\frac{1}{q\left(t\right)}\frac{\partial^{2}q\left(t\right)}{\partial t^{2}}.$$

The above function become equal if and only if they are equal to a constant ($-\omega^{2}$). Taking a positive constant, the response grows exponentially and make the system unstable:

(A5) $$-\frac{EI}{\rho A}\left(\frac{1}{\phi \left(x\right)}\right)\frac{\partial^{4\;\phi \left(x\right)}}{\partial t^{4}}=\frac{1}{q\left(t\right)}\frac{\partial^{2}q\left(t\right)}{\partial t^{2}}=-\omega^{2}.$$

Hence,

(A6) $$\frac{\partial^{2}q}{\partial t^{2}}+\omega^{2}q=0,\;q\left(t\right)=C_{1}\sin\omega t+C_{2}\cos\omega t.$$

Furthermore,

(A7) $$\frac{\partial^{4\;\phi \left(x\right)}}{\partial t^{4}}-\frac{\rho A}{EI}\omega^{2}\phi =0,\;\mathrm{let}’s\;\mathrm{assume}\;\beta^{4}=\frac{\rho A}{EI}\omega^{2},$$

$$\frac{\partial^{4\;\phi \left(x\right)}}{\partial t^{4}}-\beta^{4}\omega^{2}\phi =0,$$

which can be rewritten as follows:

(A8) $$\phi \left(x\right)=A\sin h\beta x+B\cos h\beta x+C\sin\beta x+D\cos\beta x.$$

Substituting these solutions (Equations (A1) and (A4)) into Equation (A8)),

$$U\;(x,t)=[A\sin h\beta x+B\cos h\beta x+C\sin\beta x+D\cos\beta x\;[C_{1}\sin\omega t+C_{2}\cos\omega t].$$

The constants *C*_1_ and *C*_2_ can be obtained from initial conditions, whereas the constants *A*, *B*, *C*, and *D* are obtained from boundary conditions:

$$\mathrm{At}X=0,U\;(x,t)=>\;B=-D\mathrm{and}\;\mathrm{at}X=0,\;\frac{\partial^{2}u\left(x,t\right)}{\partial t^{2}}=0=>\;A=-C.$$

Substituting *C* and *D* in terms of *A* and *B* into $\phi \left(x\right)$,

(A9) $$\phi \left(x\right)=A(\sin h\beta x-\sin\beta x)+B\left(\cos h\beta x-\cos\beta x\right).$$

At *X* = *L*, $\phi \left(x\right)=0$,

From trigonometric identities, it is known that $\cos^{2}h\beta l-\sin^{2}h\beta l=1$ and $\cos^{2}\beta l+\sin^{2}\beta l=1$.

Therefore, 2coshβlcosβl+1=0 is the frequency equation according to Euler Bernoulli. The root of these frequency equations is $\beta l=\left(n-\frac{1}{2}\right)\pi$, *n* = 1, 2, 3… for the first, second, third mode, etc.

From Equation (A3), $\beta^{4}=\frac{\rho A}{EI}\omega^{2}$, which can be rewritten as $\omega ={\left(\beta l\right)}^{2}\;\sqrt{\frac{EI\;}{\rho AL^{4}}}$.

Therefore, the natural frequency becomes

(A10) $$f_{n}=\frac{\omega_{n}}{2\pi }.$$

## Appendix B. Equation Derivation of Maximum Deflection and Maximum Stress during Collision

From Figure 16, a shuttlecock is moved to the racquet AB and hits at Point #0. The maximum deflection is written as follows: work of moving weight, work =$\;w\left(h+y_{m}\right)$ and energy =$\frac{1}{2}mv^{2}$. Therefore, the strain energy is the sum of these two.

(A11) $$U=\frac{1}{2}py_{m}=\frac{1}{2}ky^{2}{}_{m}\;,\;\mathrm{where}\;p=ky_{m},$$

where *k* is the spring constant for a load applied at point #0. Equating work and energy,

$$\frac{1}{2}mv^{2}+w\left(h+y_{m}\right)=\frac{1}{2}ky^{2}{}_{m}=>y_{m}^{2}-2\frac{w}{k}y_{m}-\left(2\frac{w}{k}\times h+\frac{m}{k}\times v^{2}\right)=0,$$

$$y_{m}^{2}-2y_{st}y_{m}-\left(2y_{st}\times h+\frac{m}{k}\times v^{2}\right)=0,$$

where $y_{st}=\frac{w}{k}$ and $k=\frac{3EI}{L^{3}}$; therefore, $y_{st}=\frac{wL^{3}}{3EI}$.

Using a quadratic equation,

(A12) $$y_{m}=y_{st}\left\{1+\sqrt{1+\frac{2h}{y_{st}}+\frac{mv^{2}}{ky_{st}}}\right\}.$$

The maximum stress becomes $\sigma_{m}=\frac{M_{m}c}{I}$, where $M_{m}=P_{m}L_{ab}.$

$P_{m}$ can be found from

$$y_{m}=\frac{p_{m}L_{ab}^{3}}{3EI}.\;\mathrm{Therefore},\;p_{m}=\frac{3EIy_{m}}{L_{ab}^{3}},\;\mathrm{and}\;p_{m}=\frac{3EIy_{m}}{L_{ab}^{2}}.$$

Finally,

(A13) $$\sigma_{m}=\frac{3Ey_{m}r_{o}}{L_{ab}^{2}}.$$
